# Evaluation of variability in cell-free DNA extraction efficiency from plasma and urine and spike-in normalization

**DOI:** 10.1038/s41598-025-06563-z

**Published:** 2025-07-02

**Authors:** Fanny Sandberg, Nicholas Kueng, Carlo Rodolfo Largiadèr, Ursula Amstutz

**Affiliations:** 1 Graduate School for Cellular and Biomedical Sciences, University of Bern, Bern, Switzerland; 2 Department of Clinical Chemistry, Bern University Hospital, University of Bern, Bern, Switzerland

**Keywords:** Cell-Free DNA, Extraction efficiency, Spike-in, Liquid biopsy, Pre-analytics, Biological techniques, Genetic techniques, PCR-based techniques, Biomarkers, Diagnostic markers, Medical research, Biomarkers, Diagnostic markers

## Abstract

Cell-free DNA (cfDNA) is a promising new biomarker for clinical use in e.g. oncology and transplantation medicine. Urinary cfDNA is also gaining interest as a non-invasive biomarker. However, cfDNA extraction is not standardised, leading to a variety of different methods being used with varying efficiencies and size-specificities. In this study, we aimed to assess the variability in cfDNA extraction efficiency for multiple cfDNA extraction methods (QIAamp Circulating Nucleic Acid Kit, Zymo Quick-DNA Urine Kit, Q Sepharose protocol (Qseph)) using the artificial spike-in CEREBIS and compare the contribution of variable extraction efficiency to overall variability in cfDNA quantities determined using droplet digital PCR (ddPCR). We found reproducible extraction efficiencies specific for each method with 84.1% (± 8.17) in plasma, 58.7% (± 11.1) for Zymo, as well as 30.2% (± 13.2) for Qseph based on the 180 bp CEREBIS (Construct to Evaluate the Recovery Efficiency of cfDNA extraction and BISulphite modification) spike-in. Additionally, while the largest proportion of the technical variability was observed between extractions, it was almost negligible compared to the biological variability. Normalization of urinary cfDNA using creatinine in urine reduced the variability, whereas when normalizing for CEREBIS-based extraction efficiency this was not consistently observed. With overall relatively consistent extraction efficiencies within each cfDNA extraction method, normalization for extraction efficiency using CEREBIS thus did not show a clear benefit but might be considered for comparisons between extraction methods.

**Supplementary Information:**

The online version contains supplementary material available at 10.1038/s41598-025-06563-z.

## Introduction

Cell-free DNA (cfDNA) has emerged as a biomarker in the medical field and belongs to the group of “liquid biopsies”. It is present in blood, urine, and other body fluids and offers a less invasive approach compared to a conventional tissue biopsy. The main potential of cfDNA lies in the non-invasive testing for physiological and pathological conditions such as prenatal screening, cancer, and organ transplant monitoring^1–3^. Given these clinical applications, interest in the extraction methods of cfDNA as a crucial step affecting the quality of downstream analyses and diagnostic accuracy is growing^4^.

The release mechanism of cell-free DNA into the bloodstream or other bodily fluids is mainly due to apoptosis or necrosis of cells^2^. An increase in cfDNA can be found in pathological processes compared to healthy individuals^5^. In addition to the released quantities of cfDNA the fragmentation pattern can give insight into its origin^6,7^. In plasma of healthy volunteers the most abundant fragment size of cfDNA was measured at around 167 base pairs (bp), which corresponds to the length of DNA wrapped around a nucleosome and an additional linker sequence^6,7^. Urinary cell-free DNA (ucfDNA) was found to have a different fragmentation pattern with the majority of fragments being shorter (80 to 112 bp)^6^. Additionally, it was shown that smaller cfDNA fragments were present with size decreasing in 10 bp steps in both plasma and urine with this effect being more pronounced in urinary samples^6^. Cell-free DNA fragments shorter than 100 bp seem to be mainly single-stranded instead of double-stranded^8^ and can be lost during downstream analysis protocols due to size-selective steps.

Currently, a wide range of methods are used for cfDNA extraction with different strategies utilising silica-based membranes, magnetic beads, and quaternary ammonium anion exchange resin^9,10^. Each method has a different range of size-selectivity, input volume, purity, and expected yield. As a consequence, extraction efficiency has been shown to vary between extraction methods and different fragment lengths for both plasma and urine^9,10^. However, particularly in light of the different characteristics of cfDNA in different body fluids, more in-depth evaluation of these extraction methods is needed to provide insights into the variability within and between samples as well as between different methods to increase our understanding of preanalytical sources of variation.

Quantification of cfDNA commonly uses fractional abundance relative to the total cfDNA, but can also be performed using absolute quantities (copies (cp.)/mL body fluid) when using suitable quantification methods. For accurate measurements of absolute cfDNA quantities, variation can occur due to preanalytical factors such as collection tubes, storage conditions, and extraction methods^11^. The determination of cfDNA extraction efficiency could thus be a useful way to account for molecules lost during extraction and minimise preanalytical variability.

One area, for which absolute quantification of cfDNA is of interest, is donor-derived cell-free DNA (dd-cfDNA), which has been proposed as a marker for allograft rejection^12,13^. Most studies conducted on dd-cfDNA are using fractional dd-cfDNA, which is increased in patients with allograft rejections. However, absolute quantities of dd-cfDNA have been suggested to increase the diagnostic accuracy^3,14^.

The evaluation of extraction efficiency is often done using non-human DNA (e.g. bacterial, plant) spiked into samples^11,15^. The spike-in used should reflect cfDNA as closely as possible in size to account for the size-specificity of extraction methods. Using a non-human, artificially synthesized DNA construct, which is not used as a control in other contexts in the laboratory, reduces the risk of contamination from other sources^16^. Evaluation of recovery efficiency from multiple analytical steps, e.g. droplet digital PCR (ddPCR) and bisulphite conversion, using only one spike-in is advantageous^11^. CEREBIS is an acronym for “Construct to Evaluate the Recovery Efficiency of cfDNA extraction and BISulphite modification” and was designed by Goh et al.^11^. It was designed as a synthetic, non-human DNA fragment with a similar size as mononucleosomal cfDNA as well as cytosine-free regions to evaluate bisulphite modification recovery^11^.

In this study, we aimed to investigate potential sources of technical variability and their contribution to variability in total yield for cfDNA extraction from plasma and urine, as well as to evaluate CEREBIS as a spike-in to determine and adjust for differences in extraction efficiency. For plasma, our study focused on the QIAamp Circulating Nucleic Acid Kit extraction method because it is widely used to extract cfDNA and showed high recovery efficiency^17^. Furthermore, using this method also enabled a comparison of the CEREBIS spike-in for measuring extraction efficiency with published data. Given the more variable fragment size distribution of cfDNA in urine compared to plasma and previous studies reporting differences in size selectivity between extraction methods^18^two cfDNA extraction methods were used (Zymo Quick-DNA Urine Kit and an in-house protocol using Q Sepharose) with the Q Sepharose protocol increasing the yield for shorter fragments^18,19^. With this assessment of important sources of technical variability in recovered cfDNA quantities, this study provides further insights into the reproducibility of standard protocols for cfDNA extraction and thus contributes to improving cfDNA-based diagnostics.

## Results

### Q Sepharose method validation

The performance of the in-house Q Sepharose-based extraction method (Qseph) was evaluated through the relationship between urinary input volume, cfDNA yield, size distribution of cfDNA, and copies of the autologous reference gene *AGO1* measured with ddPCR. A significant positive correlation was observed between the urinary input volume (10–50 mL) and the total cfDNA yield (*r* = 0.995, *p* < 0.001, Supplementary Fig. S1). Similarly, we found a strong linear correlation between the urinary input volume and the total *AGO1* copies of the eluate (*r* = 0.999, *p* < 0.001). The size distribution pattern was independent of urinary input volume (Supplementary Fig. S2).

### Yield and size distribution of extraction methods

**Fig. 1:**
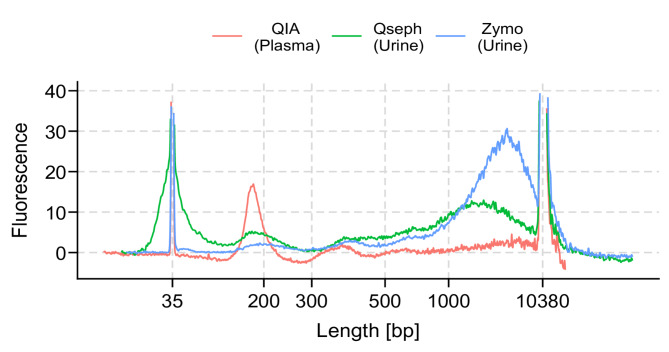
Fragment size distribution for one sample each extracted from plasma using QIA and urine samples using Zymo and Qseph. The same sample is shown for both urinary cfDNA extractions. The expected size range for Zymo is 100 bp – 23 kb and 25–23 kb for Qseph. Fluorescence intensities at a given size are not comparable between methods, due to variable input used. This graph was capped at fluorescence intensity of 40 to increase the details and the full figure is shown in the Supplementary Fig. S6.

The size distribution of cfDNA in healthy volunteers extracted from plasma using the QIAamp Circulating Nucleic Acid Kit (QIA) and urine using Qseph and the Quick-DNA Urine Kit (Zymo) showed the expected cfDNA fragment lengths (Fig. 1). The overall fragment size distribution was consistent for all three methods when measured with multiple independent samples (Supplementary Fig. S3). In more detail, plasma cfDNA had a homogeneous size distribution with a peak at around 170 bp (166–180 bp) in all samples. Urinary cfDNA showed a more variable size distribution, both between extraction methods and between biologically different samples. Extractions performed with Qseph showed a larger proportion of fragments < 90 bp and less high molecular weight DNA (> 1,000 bp) compared to Zymo as shown in Fig. 1 and Supplementary Fig. S3 and Fig. S4. Similarly, for Qspeh the proportion of fragments was higher at 89 bp than at 180 bp, whereas for Zymo this difference was less pronounced (Supplementary Fig. S4). This different size selectivity was furthermore shown in extraction experiments with a DNA ladder ranging from 50 bp to 1,000 bp, where we could only detect 50 bp fragments with Qseph but not for Zymo (Supplementary Fig. S5). The mean yield was higher for urine extracted with Zymo than with Qseph (shown in Table 1). The concentration of one extraction of Qseph was below the detection limit of Qubit (lower limit of quantitation 0.1 ng), resulting in a yield estimate of 0 ng.

**Table 1:** CfDNA concentration for each method in ng/ml plasma resp. urine.

	Source	*N*	Min	Max	Mean	SD
QIA	Plasma	36	4.93	12.0	8.72	2.06
Qseph	Urine	35	0.00	11.1	2.35	2.78
Zymo	Urine	24	0.165	22.3	3.72	5.81

Excluding the sample with a yield below 0.1ng resulted in a mean of 2.42 (SD = 2.79) for Qseph.

### Contributions to total variance in CfDNA quantity

For the first experiment (technical setup), pooled plasma samples from male volunteers were extracted by three different operators to determine the inter-operator variability. The extractions were performed by each operator on three different days to assess inter-day variability, each with three different aliquots of the plasma pool (for the estimation of inter-extraction variability). Quantification by ddPCR was performed in triplicates to determine the intra-extraction variability of the measurement method (Supplementary Fig. S7). The largest proportion of variance in the plasma technical setup was attributed to variability within the ddPCR measurement triplicates, followed by the intra-day/inter-extraction variability as shown in Table 2.

**Table 2:** Plasma pool variability proportion for measured cp/ml displayed for three different assays (target genes) within the technical setup.

Variance components	AGO1 [% (DF)]	RPP30 [% (DF)]	SRY [% (DF)]
Inter-operator	0.00 (2)	4.53 (2)	0.00 (2)
Intra-operator/Inter-extraction day	25.63 (6)	19.68 (6)	9.70 (6)
Intra-extraction day/Inter-extraction	28.28 (18)	28.48 (18)	23.45 (18)
Intra-extraction	46.09 (54)	47.31 (54)	66.86 (54)

Each assay was measured *n* = 81 in total. The fractions from the nested ANOVA were calculated using normalised absolute values. (DF = degrees of freedom).

Inter-individual variability in cfDNA yield was investigated in a second experiment (biological setup) using plasma samples from male volunteers without pooling the samples. In this setup the samples were spiked with the 180 bp CEREBIS fragment (CER180bp) and each extracted in triplicates to determine the inter-extraction variability and quantified in triplicates to evaluate the intra-extraction measurement differences (Supplementary Fig. S7). For urine extractions, biologically different samples from female and male volunteers were quantified, without pooling the samples, using the 180 bp CEREBIS fragment (CER180bp; CER-Long setup) for spiking. Additionally, in the CER-Combination setup, CER180bp as well as a shorter CEREBIS fragment at 89 bp (CER89) were added before the extraction (combination of both spike-ins), as shown in Supplementary Fig. S8. Within each setup of biologically different samples, the inter-individual variability comprised the largest proportion of variation in both plasma and urine extractions, whereas the proportion of inter- and intra-extraction variability was substantially lower within these setups (Table 3). In urine samples extracted with Zymo a larger amount of variance was attributed to differences between sexes compared to Qseph.

**Table 3:** Fraction of variance component for each setup with samples from different individuals.

	Plasma	Urine			
	Biological setup	CER-Long setup		CER-Combination setup	
Spike-in	CER180bp	CER180bp		CER89bp + CER180bp	
Variance components	QIA [% (DF)]	Qseph [% (DF)]	Zymo [% (DF)]	Qseph [% (DF)]	Zymo [% (DF)]
Inter-sex	n/a*	3.59 (1)	10.92 (1)	0.00 (1)	14.49 (1)
Intra-sex/Inter-individual	84.55 (2)	96.17 (4)	89.03 (4)	93.51 (4)	85.23 (4)
Intra-individual/Inter-extraction	6.76 (6)	0.16 (11)	0.03 (12)	5.25 (12)	n/a*
Intra-extraction	8.68 (18)	0.08 (34)	0.023 (36)	1.24 (90)	0.27 (30)

Only RPP30 measurements were included for comparability. The fractions from the nested ANOVA were calculated using normalised absolute values. (*) This component was not determined due to the study design. (DF = degrees of freedom).

### Extraction efficiency

Known quantities of CER180bp were spiked and measured to determine the extraction efficiency at 180 bp (EE180), and at 89 bp using CER89bp (EE89) in the urine CER-Combination setup. The extraction efficiency (EE) at 180 bp was similar for all plasma extractions with a mean of 84.1% (± 8.17; *n* = 36) (Table 4). Similarly, extraction efficiencies at 180 bp were reproducible for both Qseph and Zymo extractions, with mean values of 30.2% (± 13.2; *n* = 35) and 58.7% (± 11.1; *n* = 24), respectively. We found higher extraction efficiencies at 89 bp for both urinary extraction methods with 38.7% (± 16.9; *n* = 18) for Qseph and 64.6% (7.60; *n* = 6) for Zymo (Supplementary Fig. S9). For the experiments with a spiked DNA ladder and extraction efficiency estimated using Bioanalyzer, Qseph extracted shorter fragments with a higher efficiency, while this was not observed for Zymo (Supplementary Fig. S10).

**Table 4:** Mean extraction efficiencies calculated using CEREBIS spike-in fragments at 180 bp–89 bp length.

	Plasma		Urine			
	QIA		Zymo		Qseph	
Extraction efficiency	Technical setup [% ± SD]	Biological setup [% ± SD]	CER-Long setup [% ± SD]	CER-Comb. setup [% ± SD]	CER-Long setup [% ± SD]	CER-Comb. setup [% ± SD]
EE89	–	–	–	64.53 ± 7.60	–	38.69 ± 16.86
EE180	84.20 ± 8.92	83.76 ± 5.78	57.91 ± 11.23	61.12 ± 11.37	32.41 ± 15.23*	28.11 ± 10.89

(-) This value was not measured. *One sample in the CER-Long setup had a very low EE180 of 0.5%, if this sample was excluded the mean was increased to 34.4% ± 13.2.

### Adjustment of CfDNA cp/ml

To assess whether inter-extraction and overall variability can be reduced by normalization for extraction efficiency, the measured concentrations of cfDNA were divided by the extraction efficiency estimated by CEREBIS (EE180, or EE89 where applicable) and compared to the variability proportions in unadjusted quantities using ANOVA. For the study design including inter-individual variability, urine samples were additionally adjusted for urinary creatinine as a measure of urine concentration.

#### Plasma samples

**Fig. 2:**
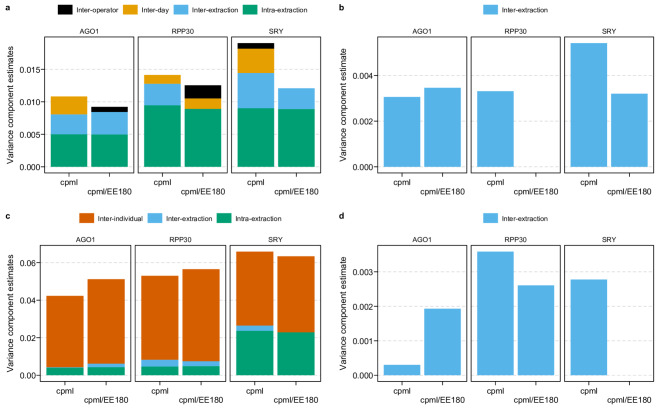
Variance component estimates of ddPCR-based cfDNA quantities in plasma samples with and without extraction efficiency adjustment. The stacked bar plots represent the total variance component estimates from the nested ANOVA for each assay in the technical plasma setup (**a**) and the biological plasma setup (**c**) with the colours indicating the nested factors. Only the variance component estimate of the inter-extraction variability is shown in (**b**) for the technical plasma setup and (**d**) for the biological plasma setup. The results using the cp/mL as well as the CER180bp extraction efficiency adjusted cp/mL (cpml/EE180) are shown.

cfDNA concentrations (cp/mL) were adjusted for extraction efficiency, determined using CEREBIS, and its effect on the variability across different experimental setups was evaluated. Adjustment of the cfDNA cp/mL using EE180 resulted in a reduction of total variability in all three assays for the technical setup (Fig. 2). In the same setup the inter-extraction variability was reduced for the *SRY* and *RPP30* measurements when accounting for extraction efficiency.

In the biological setup for plasma, the total variance was only reduced in measurements with *SRY* when adjusting for extraction efficiency determined by CER180bp (Fig. 2). When considering only the inter-extraction variability in the setup using biologically different plasma samples, a reduction in variability was observed for both *RPP30* and *SRY* (Fig. 2).

#### Urine samples

**Fig. 3:**
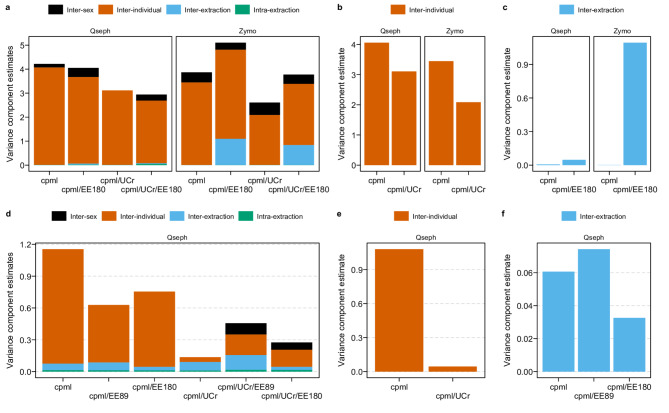
Nested ANOVA results from urine samples with adjustment for extraction efficiency and/or urinary creatinine. The variance component estimates are shown for the complete model of the urine long setup (**a**) with the nested factors distinguished by colour. Inter-individual (**b**) as well as inter-extraction (**c**) variance component estimates are shown for the urine long setup. The graphs are separated by extraction method and the analyses for cp/mL unadjusted, and adjusted for creatinine (cpml/UCr), extraction efficiency (cpml/EE) or both (cpml/Ucr/EE) are depicted. Total variance component estimates for the urine combination setup are shown in (**d**) and the estimates for the inter-individual (**e**) as well as inter-extraction (**f**) variability are shown separately. Only samples extracted with Qseph are shown for the urine combination setup with the respective extraction efficiency (EE) and/or urinary creatinine (UCr) adjustments.

The impact on the variance of cfDNA quantity was evaluated by adjustment for either EE180, EE89, urinary creatinine concentrations (UCr), EE180 and UCr, or EE89 and UCr. Adjustment with UCr resulted in a reduction of total variability in cfDNA cp/mL, and more specifically the inter-individual variability in both urinary setups for both the Qseph and Zymo extraction methods Fig. 3.

The total variance was consistently reduced in the Qseph method with adjustment for extraction efficiency (both using CER89bp- and CER180bp-based extraction efficiencies). Conversely, we observed an increase of total variability for the Zymo method after adjusting for EE. On the other hand, the inter-extraction variability based on EE-adjusted cp/mL showed no consistent reduction of variability compared to the unadjusted cp/mL.

## Discussion

In this study we analysed the variability of cfDNA yield for different extraction methods using both plasma and urine samples, and found the inter-extraction technical variability contribution to be marginal compared to the inter-individual biological differences. Reproducible extraction efficiencies were observed for the same methods across multiple samples from different donors. Other studies have shown similar ranges of recovery efficiency for the QIA method^17^ and Goh et al.^11^ had similar results using CEREBIS. The low technical variability as well as the stable recovery efficiencies suggest that the methods are robust enough to study biological differences between as well as changes within individuals.

Urine samples can be acquired non-invasively at large volumes. However many cfDNA extraction kits on the market only utilize a small input amount of ≤ 10 mL urine^18^. Increasing the input volume of urine increases cfDNA yield, providing a more accurate representation of the ongoing processes and reducing sampling bias. Both urinary extraction methods tested in this study allowed for large input volumes (up to 40 mL). The extracted fragment size distribution predominantly showed shorter fragments when using Qseph, whereas the distribution of fragments was more similar to that in plasma for Zymo. Although we did not specifically quantify single-stranded cfDNA, the increased proportion of < 80 bp fragments in Qseph may indicate a larger amount of singe-stranded cfDNA extracted, potentially allowing for downstream analyses using single-strand DNA methods^8^. This difference in efficiency between urinary extraction methods might be due to the DNA-binding beads and columns or the different composition of the buffers used for each method. Oreskovic et al.^9^ hypothesised that the increased extraction of short fragments might be due to the preconcentration of the sample with Q Sepharose and washing before applying it to a silica column. The Zymo kit, on the other hand, collects the initial DNA with clearing beads before loading them onto a silica-based column^17^. The lack of 50 bp fragments extracted using Zymo may be the result of such short fragments being lost during this step.

Furthermore, we observed higher extraction efficiencies for both 89 bp and 180 bp fragment lengths in Zymo compared to Qseph in urine. Interestingly, Qseph performed better in the PBS (phosphate buffered saline) extractions spiked with DNA ladder. The composition of urine might thus negatively influence the overall efficiency of Qseph. Although both extraction methods showed 89 bp fragments to be present in the size distribution profiles, for Qseph a more pronounced relative difference in molarity between 89 bp and 180 bp was observed, reflecting the over 30% higher extraction efficiency at 89 bp compared to 180 bp for this method. Even though no ddPCR-based estimation of extraction efficiency was performed for fragments shorter than 89 bp, the observed size distribution profiles suggest that for downstream applications targeting ultra-short ucfDNA fragments, using Qseph might be more suitable, whereas Zymo should be considered for higher yields^8,11^.

In the technical setup the inter-extraction variability comprised about 25% of the total variability in cfDNA yield. However, in a more realistic setting including biologically different samples, the relative contribution of inter-extraction variability to total variability was strongly reduced and almost 90% of variability was attributed to inter-individual differences. Adjustment for extraction efficiency did not consistently reduce the inter-extraction variability, including urinary cfDNA extractions involving a more variable size distribution and shorter cfDNA fragments. This leads us to conclude, that although the use of the CEREBIS spike-in may provide an estimate of the overall recovery efficiency of an extraction method, this approach is not accurate enough to adjust for the relatively minor inter-extraction variability. Nevertheless, this spike-in approach proved relevant for comparing extraction efficiencies between methods. Furthermore, the incorporation of a shorter spike-in using the same original DNA construct made it possible to assess and compare size-specific recovery efficiencies between methods. Given the variability in size distribution of ucfDNA and the variable size selectivity of different downstream protocols such as library preparation methods or ddPCR amplicon sizes, knowledge of size-specific recovery efficiencies for a given cfDNA extraction method is relevant for ucfDNA.

Variability in urinary cfDNA measurements was shown to be reduced after normalization with creatinine measured in urine. Our findings thus indicate that accounting for urine concentration in this manner may allow for better comparability between samples and individuals. Creatinine can be measured at relatively low cost, making it a cost-efficient adjustment parameter. Additionally, in some clinical contexts such as kidney transplantation, urinary creatinine is measured as part of the clinical routine and thus readily available without extra cost.

The strength of this study was the systematic comparison of the effects of technical and biological factors on the efficiency of different cfDNA extraction methods, as well as allowing for an assessment of the benefit of measuring and adjusting for the extraction efficiency using CEREBIS. The original study introducing CEREBIS by Goh et al.^11^ did not include inter-individual or inter-extraction variability of the EE calculated with CEREBIS. Therefore, this independent evaluation of the spike-in provides further insight into its benefits and disadvantages. The limitations of our study include the use of only one extraction method for plasma, and that only two sizes of spike-ins were used. A wider range of spike-in sizes might be more informative regarding the size selectivity of extraction methods, particularly for urine, but would be analytically more labour and cost intensive. Additionally, analysis of extracted cfDNA using single-stranded library preparation and high-throughput sequencing could provide additional insights into the size distribution and size selectivity of different ucfDNA extraction methods. In this context, although CEREBIS approximates cfDNA, it does not have jagged ends like cfDNA and only accounts for double-stranded molecules^7^. Single-stranded DNA sequencing of cfDNA extracts in future studies could thus also provide insight into the efficiency of different extraction methods for extracting jagged-ended or single-stranded cfDNA. Another limitation was the exclusive use of samples from healthy volunteers. This provided a baseline for analysing the technical variability of the methods, but patient samples should also be taken into consideration for further systematic evaluations as they may differ in cfDNA quantity and fragment size distribution. Finally, we did not account for nuclear genomic DNA contamination of samples, e.g. by measuring a longer PCR amplicon^3^.

In conclusion, we found no consistent reduction of the inter-extraction variability when accounting for the extraction efficiency using CEREBIS for plasma or urine samples from different individuals. While CEREBIS-based adjustment for extraction efficiency did not appear to provide a benefit for increasing the comparability between biological samples, spike-ins with different fragment sizes can be valuable in the evaluation and comparison of different extraction methods. Finally, the minor contribution of the technical variability to the total variability suggests that technical differences between extractions using the same method are negligible as confounders for the quantification of cfDNA.

## Methods

### Sample collection and ethics statement

Healthy volunteers were recruited according to the following criteria: (1) > 18 years old, (2) no pregnancy event three months prior to sample collection, (3) no organ, bone marrow, or stem cell transplantation received, (4) never received a blood transfusion and (5) generally healthy. All volunteers were recruited at Inselspital (Bern University Hospital, Switzerland) between May 2022 and April 2024. Written informed consent was obtained from each healthy volunteer and the study was approved by the ethics committee of the Canton of Bern, Switzerland (2020 − 00953), and conducted in accordance with its guidelines and regulations. All samples were processed within 2 h after collection.

Blood samples from six healthy male volunteers were collected in Sarstedt S-Monovette^®^ 7.5 mL K3E (Sarstedt, Nümbrecht, Germany) and centrifuged at 2,000x g for 15 min at room temperature (RT). The plasma was additionally centrifuged at 3800x g for 10 min at RT and the plasma supernatant was collected. All individual plasma samples were stored at −80 °C until they were combined into a single pool. From the plasma pool a total of 27 aliquots of 5 mL plasma each was prepared and stored at −80 °C until extraction. Additionally, blood samples from three healthy male volunteers were collected and processed in the same way as described above, but were not pooled and instead stored individually at −80 °C.

Urine samples from healthy volunteers were collected on two occasions (each time from three male and three female volunteers) in 50 mL LoBind^®^ tubes (Eppendorf SE, Hamburg, Germany) with 2.5 mL of Streck^®^ Urine Preserve (Streck Inc., La Vista, NE, USA) added for nucleic acid stabilisation. After centrifugation at 2,000x g for 15 min at RT the urine supernatant was stored at −80 °C until extraction. Creatinine concentration (UCr) was measured in each urine sample.

### Spike-in

The CEREBIS spike-in was chosen for its 180 bp length, which is in the range of most cfDNA in plasma (166 bp according to Markus et al.^6^). Furthermore, it is synthesised artificially, does not map to the human genome and can be used together with additional downstream processing including bisulphite modification. Additionally, a shorter fragment of the original 180 bp CEREBIS fragment (CER180bp) was designed comprised of only 89 bp of the full fragment (CER89bp) to account for shorter fragments present in urine (Supplementary Table S1). CER180bp and CER89bp stock solutions were quantified with dilution series quantified using ddPCR to determine the exact concentrations (Supplementary Fig. S11).

### CfDNA isolation

#### Plasma processing

QIA: cfDNA extraction from plasma was performed using QIAamp Circulating Nucleic Acid Kit (QIA) (Qiagen, Hilden, Germany). Each extraction was performed using 5 mL of plasma thawed to RT and spiked with 20,000 cp. of CER180bp. The extraction was performed as described in Kueng et al.^18^ but without addition of carrier RNA.

### Urine processing

Zymo: Urine samples were extracted using the Quick-DNA Urine Kit (Zymo Research, Irvine, CA, USA) according to the method described in Kueng et al.^20^ with an input volume of 20 mL. Prior to extraction the samples were either spiked with 85,000 cp. of CER180bp, or 45,000 cp. of CER180bp and 45,000 cp. of CER89bp, see study design below.

Qseph: This protocol was developed to effectively isolate cfDNA between 25 bp and 23 kb from larger volumes of urine. The method was adjusted from Shekhtman et al.^19^Oreskovic et al.^9^ and Dudley et al.^21^ as previously described^22^. cfDNA was extracted from 20 mL urine thawed at RT, spiked with CER180bp (85,000 cp.), or CER180bp (45,000 cp.) and CER89bp (45,000 cp.), mixed with 100 µL Q Sepharose Fast Flow (Merck KGaA, Darmstadt, Germany) per 10 mL urine and incubated at RT for 30 min on a rocking platform. The sample was centrifuged at 1800x g for 5 min and the supernatant discarded. The pellet was resuspended in 1 mL low-salt buffer (0.3 mol/L LiCl and 10 mmol/L NaOAc at pH 5.5), transferred to a Micro Bio-Spin Chromatography Column (Bio-Rad Laboratories Inc., Hercules, CA, USA) and filtered at 800x g for 1 min. The resin was washed 3–5 times with 670 µL of low-salt buffer (800x g, 1 min) until the resin was not blue anymore from the Streck^®^ Urine Preserve. cfDNA was eluted twice using 670 µL high-salt buffer (2 mol/L LiCl and 10 mmol/L NaOAc at pH 5.5) and the eluate transferred to 4 mL 85% ethanol. It was incrementally transferred to a QIAquick Spin Column (Qiagen, Hilden, Germany) and filtered (5,000x g, 30 s). The column was washed with 750 µL PE buffer (Qiagen, Hilden Germany), 500 µL PE buffer and 750 µL 100% ethanol (3,000x g, 30 s). After drying the column (20,000x g, 2 min) 60 µL elution buffer (Qiagen, Hilden, Germany) was added, incubated for 3 min and centrifuged at 20,000x g for 30 s. The eluate was reapplied to the matrix with an incubation time of 3 min and a final centrifugation (20,000x g, 2 min).

#### Eluate quality measurements and storage

All cfDNA eluates were weighed on an XPR225DR scale (Mettler Toledo, Greifensee, Switzerland) to determine the volume of the eluate, concentrations were measured using the Qubit 1X dsDNA HS Assay Kit and a Qubit 4 fluorometer (Thermo Fisher Scientific, Waltham, MA, USA), and stored until analysis at −20 °C.

### Validation of Qseph

The purity of the eluate was assessed using a NanoDrop™ One spectrophotometer (Thermo Fisher Scientific, Waltham, MA, USA) and the concentration was determined by Qubit as described above. Size distributions of the eluates were measured using the High Sensitivity DNA Kit with the 2100 Bioanalyzer system (Agilent, Santa Clara, CA USA). Of note, due to dilution prior to analysis in some samples to meet loading concentration 100 pg/µL – 5 ng/µL, Bioanalyzer results are not quantitatively comparable. Linearity of the increase in yield with increasing input volume was assessed by extracting three different urine samples using five different input volumes (10 mL, 20 mL, 30 mL, 40 mL, 50 mL) each. Total cfDNA concentration for all samples was measured using Qubit. One of the three series was additionally quantified by autologous (*AGO1*) copies determined using ddPCR.

Inhibition of ddPCR was detected for some Qseph eluates, resulting in reduced fluorescence signal intensity. For samples without a clear separation of the fluorescence clusters, the measurement was repeated with a two-fold dilution, which resulted in a normalization of signal intensity.

#### Size distribution of urine extraction methods

To assess the size distribution at known fragment sizes after extraction, a double-stranded DNA ladder (GeneRuler 50 bp DNA ladder, Thermo Fisher Scientific, Waltham, MA, USA) with sizes ranging from 50 bp to 1,000 bp was spiked at 200 ng into 10 mL phosphate buffered saline (PBS) pH 7.4. Three separate 10 mL aliquots of the ladder-spiked solution were thereafter extracted each with the Zymo protocol and the Qseph protocol. Bioanalyzer was used to estimate the respective concentrations using peak integration for each fragment of the ladder. The input for the Bioanalyzer consisted of 1 µL extraction eluate for Qseph, whereas the Zymo eluates were diluted by a factor 1.5 to account for the lower elution volume, and 1 µL of the diluted eluate was loaded. The mean concentration and yield of the measurement triplicates were calculated for each extraction method at every measurable ladder position. Extraction efficiency at each ladder peak position was determined by dividing the sample yield through the ladder input, which consisted of the concentration at that position multiplied by the total volume used for spiking.

### Study design

#### Plasma

For plasma extraction, two experiments were performed. The first experiment (technical setup) was performed using pooled plasma and aimed to assess inter-operator, inter-day and inter-extraction technical variability. Specifically, three operators performed cfDNA extractions of three plasma pool aliquots on three different days each. To assess technical variability in comparison to biological variability, a second experiment was performed (biological and technical setup) to comparatively assess inter-individual and inter-extraction variability. For this experiment unpooled plasma samples from three different volunteers were extracted on the same day by the same operator with triplicate extractions for each sample.

CfDNA in all samples was quantified using two autologous reference genes (*AGO1*, *RPP30*) and one Y-chromosomal gene (*SRY*), and additionally, quantification of CEREBIS was performed for each sample.

#### Urine

For urine extractions, the two extraction methods Zymo and Qseph were compared in two experiments. The first experiment used the original CEREBIS CER180bp spike-in (long setup), whereas the second experiment additionally included the shorter CEREBIS subfragment CER89bp (combination setup) to compare extraction efficiencies at two different fragment lengths. For each experiment urine samples from three female and three male volunteers were extracted with Zymo and Qseph. Extractions were performed in triplicate except for Zymo in the combination setup, where only one sample per individual was extracted as a control. The extractions were performed by the same operator on the same day.

For all urine samples cfDNA was quantified using one autologous reference gene (*RPP30*) and quantification of CEREBIS was performed by quantifying both the sequence shared by CER180bp and CER89bp (CEREBIS_short) as well as the sequence unique to CER180bp (CEREBIS_long).

### Quantification of cell-free DNA

All quantifications were performed using droplet digital PCR (ddPCR). For the autologous reference genes an assay for *AGO1* (dHsaCP2500349) from Bio-Rad (Bio-Rad Laboratories Inc., Hercules, CA, USA) was used as well as the *RPP30* assay from Härmälä et al.^23^.The *SRY* assay was used as described in Kueng et al.^20^.The ddPCR assays to measure CEREBIS were newly designed (Supplementary Table S2).

The ddPCR measurements were performed as previously described in Kueng et al.^20^ in triplicates including the subsequent adjustment for non-amplifiable fragments, which was applied to all plasma measurements except for the spike-in quantifications. Both CEREBIS assays did not show any nonspecific amplification in plasma samples without spike-in (*n* = 8). No co-hybridization of the Y-chromosomal or autologous reference gene assays was observed with CER180bp or CER89bp.

### Data analysis

#### Extraction efficiency calculation

Extraction efficiency for each sample was calculated by dividing total copies of CER180bp in the eluate by the total copies CER180bp spiked as shown in Eq. (1)

1 $$\:\text{EE180}=\frac{\text{CER180bp sample [cp]}}{\text{CER180bp spiked [cp]}}$$

Total copies (CER180bp sample) were calculated using the mean of the measurement triplicates and the volume of the eluate assessed by weight. The total copies of CEREBIS spiked (CER180bp spiked) were derived from the mean measured in triplicates of the pure spike-in solution and the volume spiked before the extraction. The EE180 was calculated using only measurements of the CEREBIS_long assay.

To account for differences between batches, a pure spike-in CER180bp aliquot was quantified with both CEREBIS assays and the total copies of CER89bp for each sample calculated (as described in Eq. (2) with the CER180bp spike-in solution measured with the CEREBIS_short assay.

2 $$\begin{aligned}\:\text{CER89bp sample [cp]}=&{\text{CEREBIS sample [cp]}}_{\text{CEREBIS short}}\\ & -{\text{CER180bp spiked [cp]}}_{\text{CEREBIS short}}\text{*}\text{EE180}\end{aligned}$$

The CEREBIS short assay detects both CER180bp and CER89bp (Supplementary Fig. S12). To identify the amount of CER89bp, the quantity of CER180bp thus has to be subtracted. The quantity in the pure CER180bp spike-in solution measured with the CEREBIS_short assay with the sample-specific EE180 was used to account for differences between batches.

The extraction efficiency for the 89 bp CEREBIS spike-in was calculated according to the Eq. (3).

3 $$\:\text{EE89}=\frac{\text{CER89bp sample [cp]}}{\text{CER89bp spiked [cp]}}$$

#### Adjustment of cp/ml

The original cp/mL were adjusted for extraction efficiency by division withEE180 and EE89, respectively. The correction for creatinine in urine was achieved by dividing the original cp/mL by UCr (µmol/L/1,000), resulting in cp/µmol Creatinine. The adjustment containing both extraction efficiency and UCr was done by dividing the UCr corrected cp/µmol with the respective extraction efficiency.

Ensuring the comparability of measurements from different assays or extraction methods required normalization by the overall mean of each assay for each setup, adjustment, and extraction method. All downstream analyses were carried out with normalized values.

#### Statistical analysis

All analyses were performed in R 4.4.1. Data visualisation was done with the R package *ggplot2* v3.5.1. Analysis of variance (ANOVA) models were performed using the *VCA* package v1.5.1. The ANOVA analysis were performed in a nested design with the factors and their respective levels depicted in the Supplementary Fig. S7 and Fig. S8.

## Electronic supplementary material

Below is the link to the electronic supplementary material.

Supplementary Material 1

## Data Availability

The original raw data supporting the conclusions of this article will be made available by the corresponding author upon reasonable request.
