# Supplementary material for: Evaluation of variability in cell-free DNA extraction efficiency from plasma and urine and spike-in normalization
Source: Sci Rep. 2025 Jul 2;15:22999. doi: 10.1038/s41598-025-06563-z (PMC12218894; doi:10.1038/s41598-025-06563-z)
Supplement: Supplementary file 1 — Supplementary Material 1 [file 41598_2025_6563_MOESM1_ESM.pdf]

Supplementary Material

Manuscript title: Evaluation of variability in cell-free DNA extraction efficiency from plasma and urine and spike-in normalization

Authors: Fanny Sandberg, Nicholas Kueng, Carlo R. Largiadèr, *Ursula Amstutz

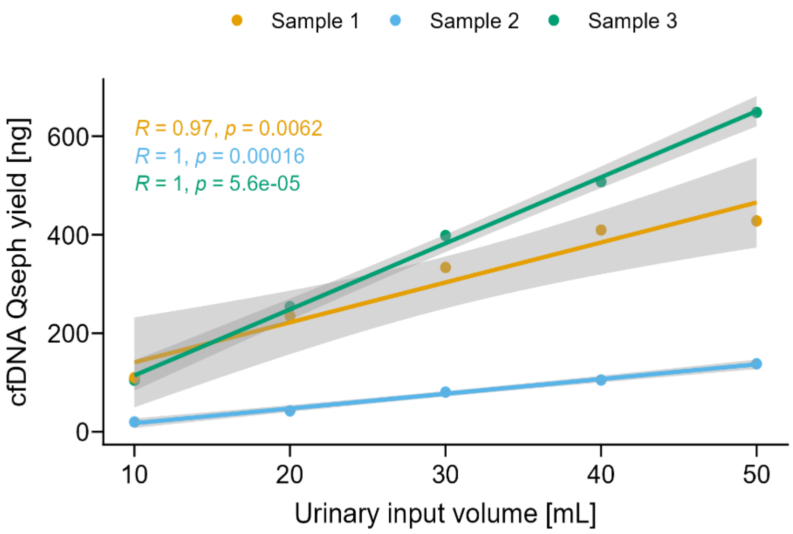

Supplementary Figure S1: Correlation of cfDNA yield after extraction with Qseph for different urine input volumes. The samples were from independent healthy individuals and the same sample was divided into aliquots to achieve the five different input volumes.

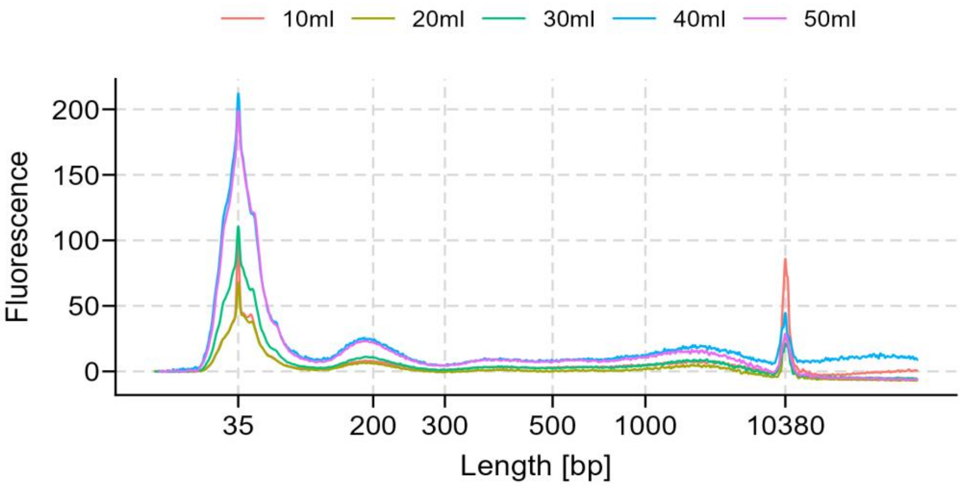

Supplementary Figure S2: Size distribution after Qseph extraction with different urine input volumes.

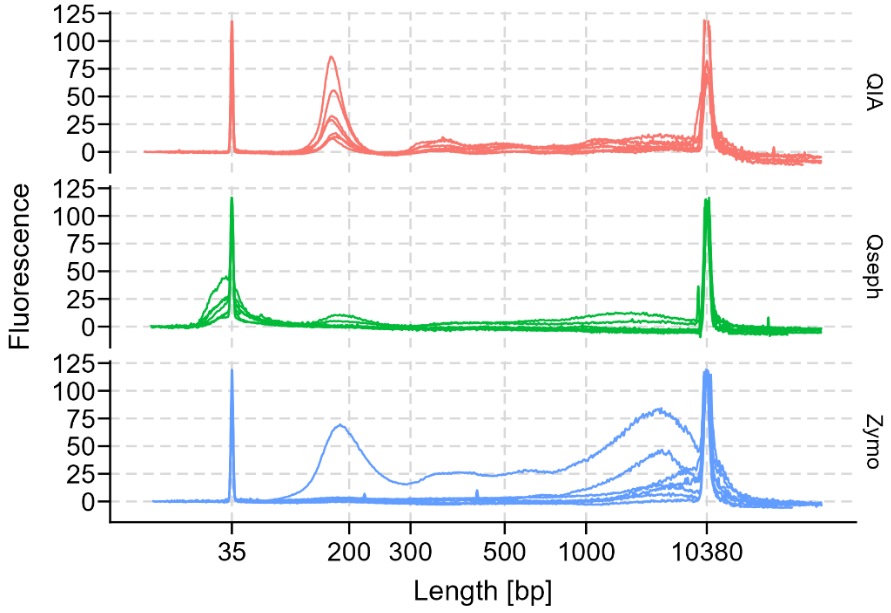

Supplementary Figure S3: Representative bioanalyzer data for each extraction method from independent healthy individuals (n=7 for each method) with the same urine extracted with both methods for each individual. The figure was capped at a fluorescence intensity of 120 to increase the details of each fragment pattern, only signal from the lower and upper markers were cut off.

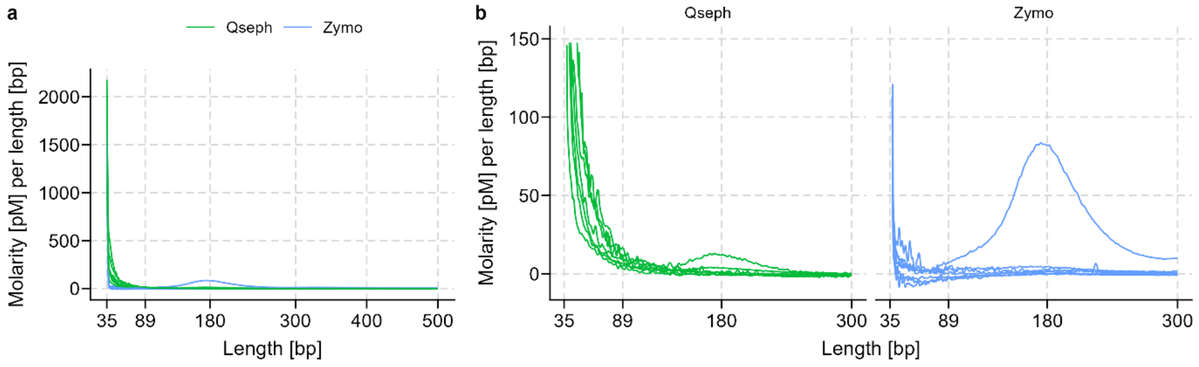

Supplementary Figure S4: Bioanalyzer spectra for short fragments with molarity per fragment length for both urinary extraction methods from independent healthy individuals (n=7 for each method). Molarity per length is shown for fragments larger than the lower marker (at 35 bp). An overview of all the samples up to 500 bp is shown in (a). For each urinary extraction method the details between 35 bp and 300 bp are shown in (b). The level of molarity between samples cannot be compared due to different input volumes used, however, the relative proportions within each sample can be evaluated.

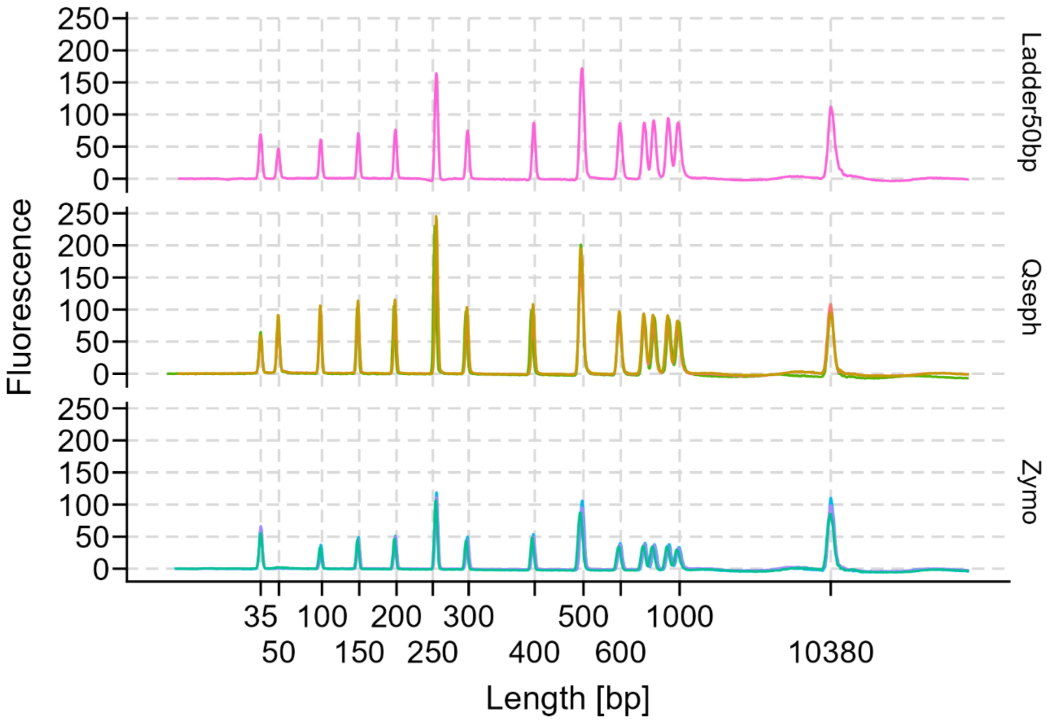

Supplementary Figure S5: Bioanalyzer results from DNA ladder spiked into PBS and extraction with Qseph and Zymo. The input for Zymo and Qseph used corresponds to the same pre-extraction volume of spiked PBS, whereas the ladder was loaded at 1 ng/µL.

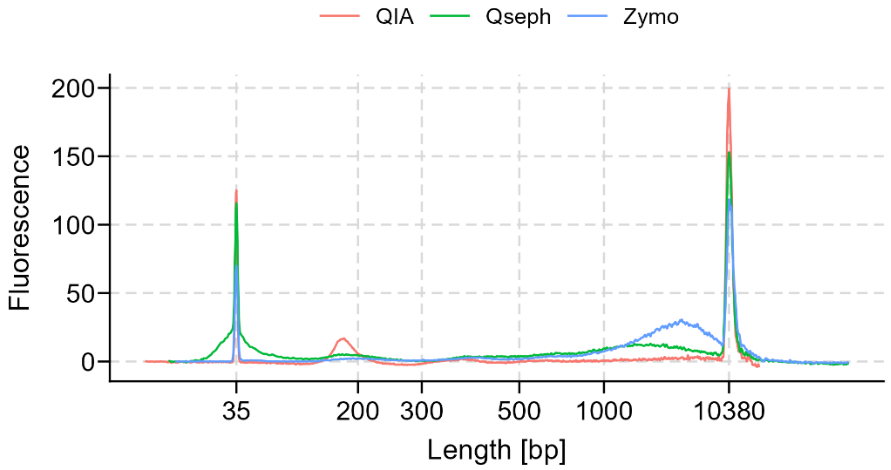

Supplementary Figure S6: Full intensity spectrum of fragment size distribution for the sample shown in Figure 1.

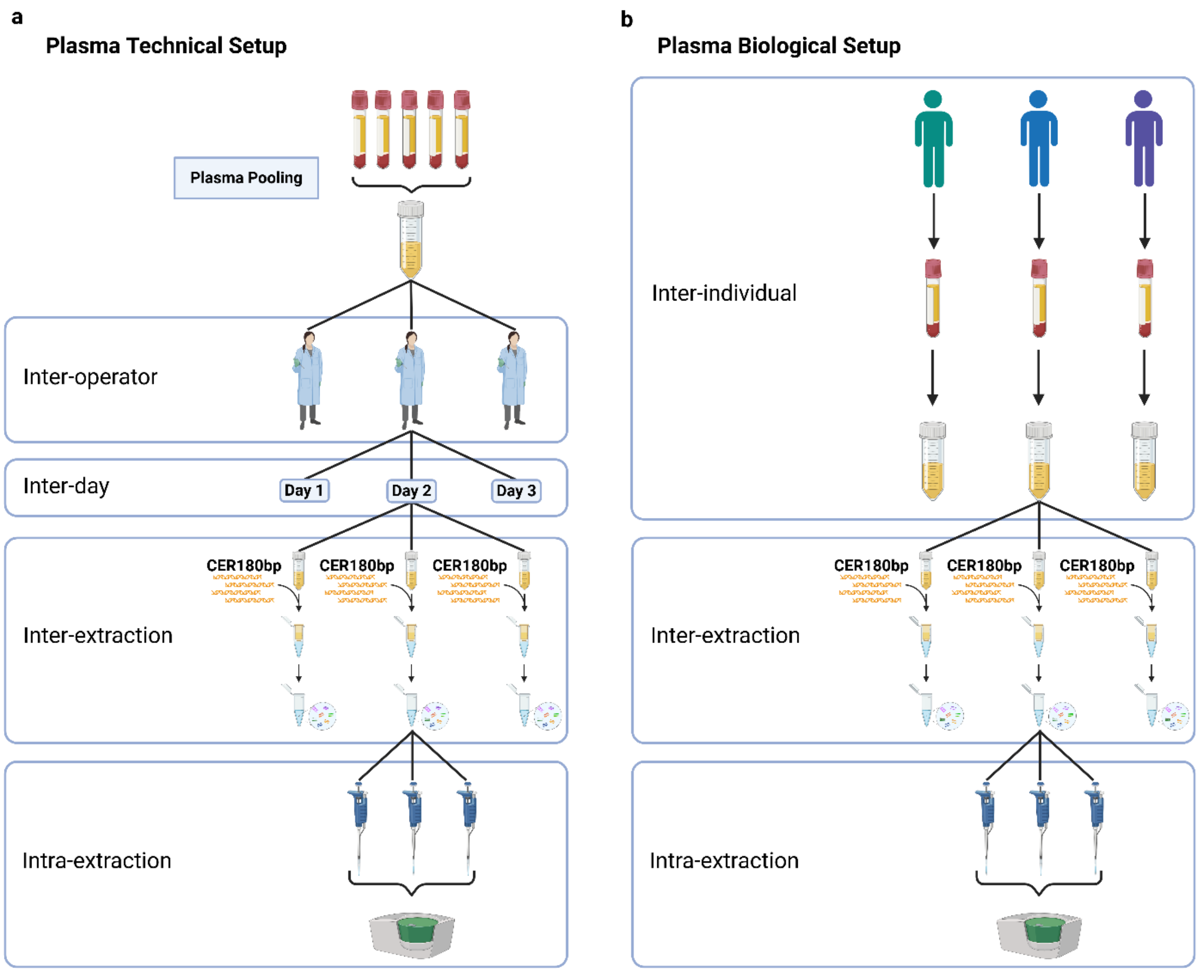

Supplementary Figure S7: Schematic illustration of the study design for the plasma samples with the levels of the nested ANOVA indicated

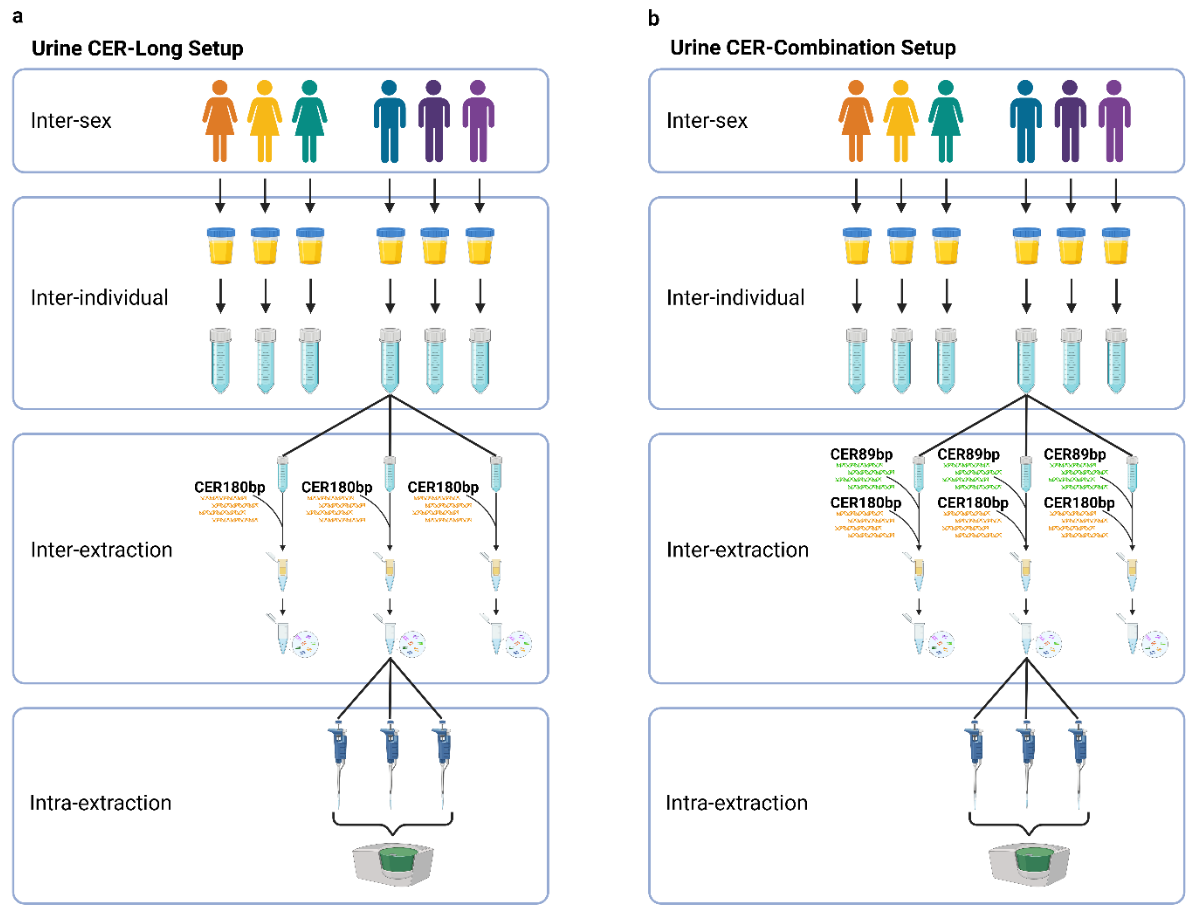

Supplementary Figure S8: Schematic illustration of the study design for the urine setups with the levels of the ANOVA results displayed

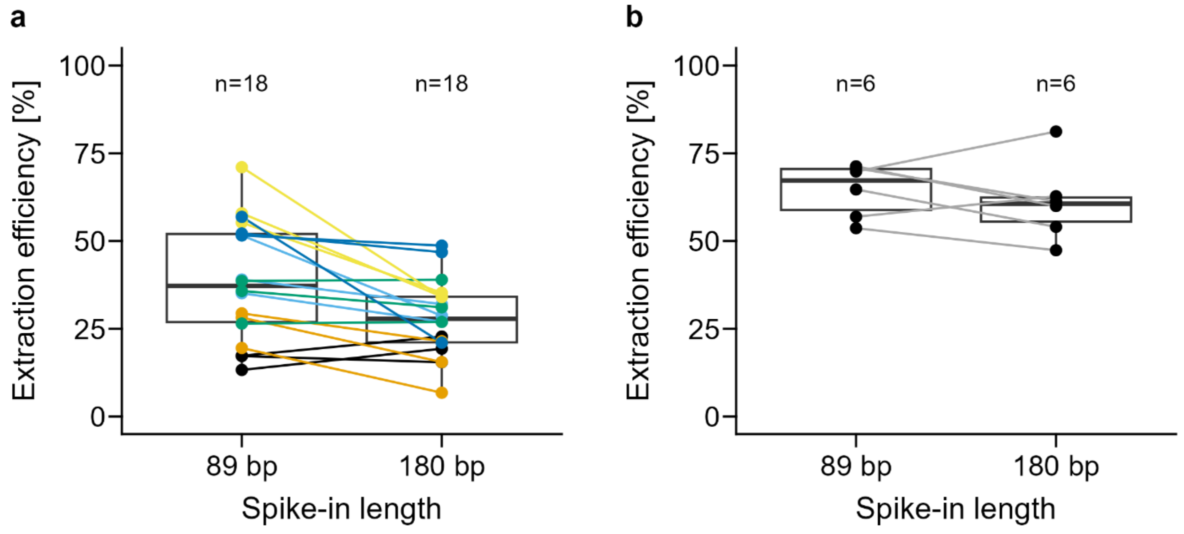

Supplementary Figure S9: Extraction efficiencies in the urine CER-Combination setup for each spike-in length. The extraction efficiencies for each sample were extracted in triplicates with Qseph (a) and once with Zymo (b). The colours represent technical replicates from the same individual.

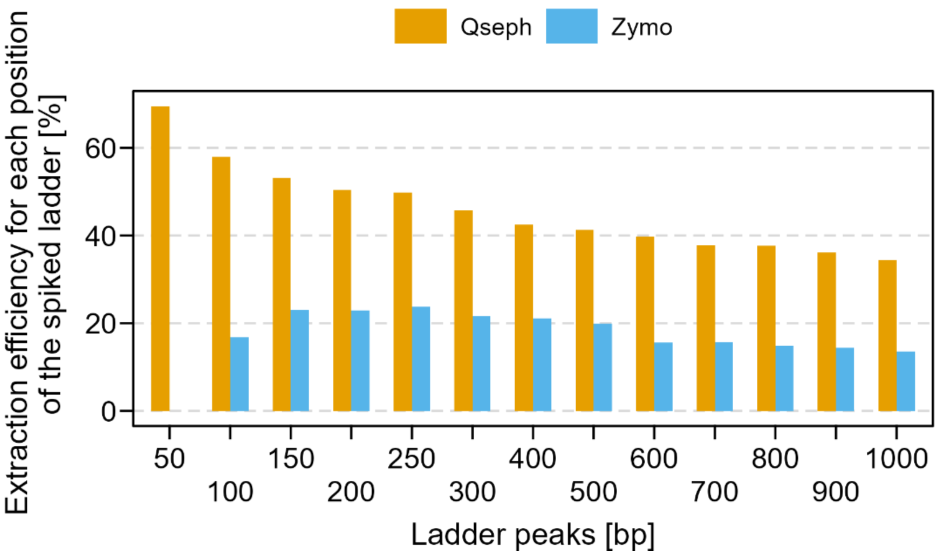

Supplementary Figure S10: DNA ladder spiked into PBS extraction efficiency calculated using concentrations estimated by Bioanalyzer peak integration. No peak was detected for Zymo at 50bp.

Supplementary Table S1: CEREBIS spike-in sequences.

CER180bp	5’-<br>GTGGGAGTGGATGAGGTTTGAGTTGGTGAGGTGTGAGTGGTAGATGTTGTGTCTGT<br>CGAGCGAGCGAGTGTAGAGTGTAGTGTCGGATGGGTGAGTTGAGATGAGATGGG<br>TAATGCGCGTGTGGTAGGAGTTGATGATAGGGTGAGAGTGGTGAAAGTTGTGAAG<br>AGTGTTGTAGGTGTA-3’
CER89bp	5’-<br>TGGATGAGGTTTGAGTTGGTGAGGTGTGAGTGGTAGATGTTGTGTCTGTCGAGCGA<br>GCGAGTGTAGAGTGTAGTGTCGGATGGGTGAGT-3’

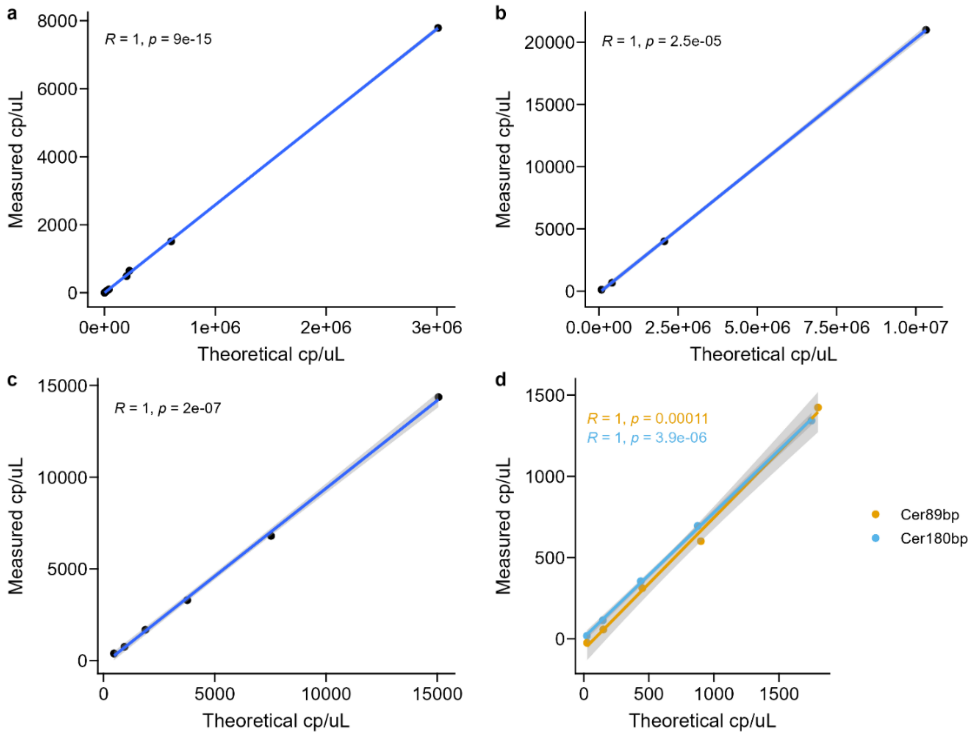

Supplementary Figure S11: Dilution series for both CEREBIS assays and both spike-ins. The dilution series for the CER180bp spike in and the CEREBIS_long assay is shown in (a) based on n=9 measurements. The same spike-in measured with CEREBIS_short assay using n=4 is depicted in (b). The dilution of CER89bp measured with CEREBIS_short assay is shown in (c) with n=6. (d) Mean calculated spike-in concentrations (n=5 each), after both spike-ins were mixed and measured with both assays.

Supplementary Table S2: ddPCR assay primer and probe sequences

Assay	Oligonucleotide	Sequence
CEREBIS_long	Forward primer	5'-AGAGTGTAGTGTCGGATGGG-3'
	Reverse primer	5'-AACTTTCACCACTCTCACCC-3'
	Probe	5'-FAM-TGAGATGGGTAATGCGCGTGTGGTAGGAGT-BHQ1-3'
CEREBIS_short	Forward primer	5'-TGGATGAGGTTTGAGTTGGTG-3'
	Reverse primer	5'-ACTCACCCATCCGACACTAC-3'
	Probe	5'-FAM-TGTGTCTGTCGAGCGAGCGAGTGTAGAGT-BHQ1-3'
RPP30	Forward primer	5'-AGATTTGGACCTGCGAGCG-3'
	Reverse primer	5'-GAGCGGCTGTCTCCACAAGT-3'
	Probe	5'-HEX-TTCTGACCTGAAGGCTCTGCGCG-BHQ1-3'
SRY	Forward primer	5'-TGTCCTACAGCTTTGTCCAG-3'
	Reverse primer	5'-CCACTTACCGCCCATCAAC-3'
	Probe	5'-FAM-ACCGCAGCAACGGGACCGCT-3'

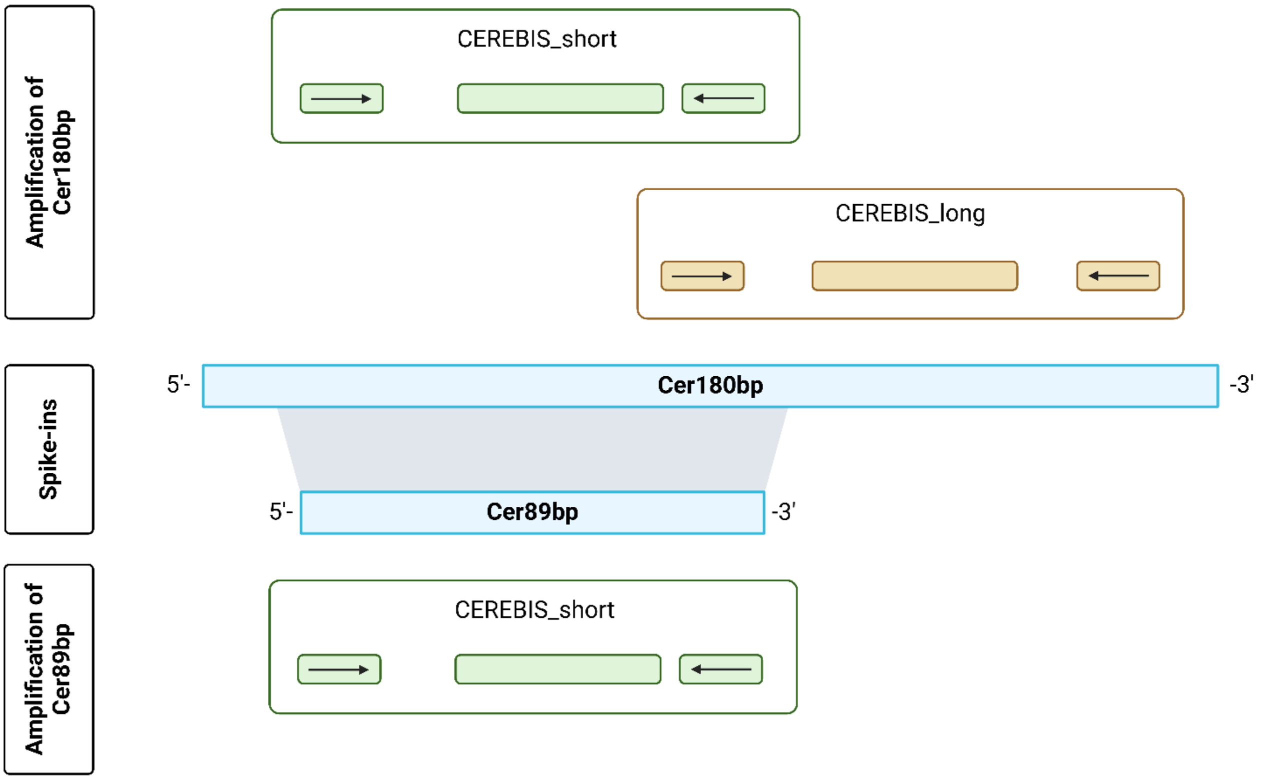

Supplementary Figure S12: Schematic illustration of the targets for the CEREBIS assays.
